# Proteome sequencing and analysis of *Ophiocordyceps sinensis* at different culture periods

**DOI:** 10.1186/s12864-020-07298-z

**Published:** 2020-12-11

**Authors:** Bo Zhang, Bo Li, Xiao-Hui Men, Zhe-Wen Xu, Hui Wu, Xiang-Tian Qin, Feng Xu, Yi Teng, Shui-Jin Yuan, Li-Qun Jin, Zhi-Qiang Liu, Yu-Guo Zheng

**Affiliations:** 1 Key Laboratory of Bioorganic Synthesis of Zhejiang Province, College of Biotechnology and Bioengineering, Zhejiang University of Technology, Hangzhou, 310014 China; 2 HuaDong Medicine (Hangzhou) Bailing Biological Technology Co., Ltd, Hangzhou, 311220 China; 3 East China Pharmaceutical Group Limited Co., Ltd, Hangzhou, 311000 China

**Keywords:** *O. sinensis* zjut, Proteome, Differentially expressed proteins, Active ingredients

## Abstract

**Background:**

*Ophiocordyceps sinensis* is an important traditional Chinese medicine for its comprehensive active ingredients, such as cordycepin, cordycepic acid, and *Cordyceps* polysaccharide. *O. sinensis* zjut*,* a special strain isolated from *O. sinensis,* has similar pharmacological functions to wild *O. sinensis*. Currently, *O. sinensis* with artificial cultivation has been widely studied, but systematic fundamental research at protein levels has not been determined.

**Results:**

Proteomes of *O. sinensis* zjut at different culture periods (growth period, 3rd day; pre-stable period, 6th day; and stable period, 9th day) were relatively quantified by relative isotope markers and absolute quantitative technology. In total, 4005 proteins were obtained and further annotated with Gene Ontology, Kyoto Encyclopedia of Genes and Genomes database. Based on the result of the annotations, metabolic pathways of active ingredients, amino acids and fatty acid were constructed, and the related enzymes were exhibited. Subsequently, comparative proteomics of *O. sinensis* zjut identified the differentially expressed proteins (DEPs) by growth in different culture periods, to find the important proteins involved in metabolic pathways of active ingredients. 605 DEPs between 6d-VS-3d, 1188 DEPs between 9d-VS-3d, and 428 DEPs between 9d-VS-6d were obtained, respectively.

**Conclusion:**

This work provided scientific basis to study protein profile and comparison of protein expression levels of *O. sinensis* zjut, and it will be helpful for metabolic engineering works to active ingredients for exploration, application and improvement of this fungus.

**Supplementary Information:**

The online version contains supplementary material available at 10.1186/s12864-020-07298-z.

## Background

*Ophiocordyceps sinensis* is an important traditional Chinese medicine and healthy food in China [1–3]. Previous studies have revealed that active ingredients of *O. sinensis,* including D-mannitol, cordycepin, purine nucleotides and polysaccharide, have various pharmacological functions, such as adaptogenic activity, immunomodulatory effects, antioxidant activity and anti-cancer [4, 5]. However, due to high demand and insufficient supply for wild *O. sinensis*, the medicinal value development has been seriously limited [6, 7]*.* Recently, several strains that isolated from wild *O. sinensis* have similar pharmacological functions to wild *O. sinensis* [8], representing an useful alternative for production. Moreover, new potential drugs and active compounds derived from natural sources have been screened as effective disease treatment from *Hirsutella sinensis,* which is the potential anamorph of *O. sinensis.* The antitumor activity of *H. sinensis* mycelium was found as seen in studies on human tissue, including prostate (PC3), breast (MCF7), hepatocellular (HepG2, Hep3B) and colorectal (HT-29) [9, 10]. Previous studies have revealed the active ingredients of artificially-cultivated *O. sinensis* had extensive medicinal value for human health, such as anti-fatigue activity [11], immunomodulatory activity [12], antioxidant activity [13] and anti-obesity effects [14]. Furthermore, the productions of active ingredients in *H. sinensis* by submerged fermentation were promoted and satisfying on the basis of genetic study [15–17].

Proteomics is the large-scale study of proteins, and it can be divided into the areas of large-scale identification of proteins and their post-translational modifications, comparison of protein expression levels, and protein-protein interactions [18]. In previous studies, biochemical methods have been widely used to study proteome, including polyacrylamide gel electrophoresis (PAGE), two-dimensional electrophoresis (2-DE) liquid chromatography, surface-enhanced laser desorption ionization-time of flight-mass spectrometry (SELDI-TOF-MS), and matrix-assisted laser desorption ionization-time of flight-mass spectrometry (MALDI-TOF-MS) [19, 20]. Recently, with the ability to carry out relative (or absolute) quantification in up to eight phenotypes, isobaric tags for relative and absolute quantification (iTRAQ) have caught the attention of proteomics community [21, 22]. iTRAQ-based proteomics has been used in studying secretome, plasma membrane proteome, and intracellular proteome [22–24].

Genome sequence of *O. sinensis* has been reported, which revealed the pathogenic mechanism during life cycle [25]. Then, transcriptomes of *H. sinensis* at diffident culture periods have been sequenced and analyzed to describe metabolic pathway and infection mechanism [26]. Meanwhile, the comparative proteomic has shown a snapshot proteome profile and revealed the similarity of the proteins and metabolites composition between naturally- and artificially-cultivated of *O. sinensis* [27]. Dong et al studied the dynamic polymorphic alterations among differentially expressed proteins of multiple intrinsic fungi in the caterpillar body and stroma of natural *O. sinensis* during maturation, and the results revealed there were the apparent proteomic polymorphism dissimilarity of these organisms to support the integrated micro-ecosystem hypothesis for natural *O. sinensis* [28]. In addition, there were some non-quantitative studies about the proteomics of *O. sinensis* [27–30]. In fact, without the complement of proteomics, only genome and transcriptome sequences are not sufficient to elucidate biological functions. Moreover, there is no strict linear relationship between genes and the corresponding proteins [18]. Therefore, proteome of the *O. sinensis* zjut was used to the large-scale study of gene functions directly at the protein level and tried to illuminate the synthesis mechanism of active ingredients in this study.

In this study, proteomes of the *O. sinensis* zjut at different culture periods were relatively quantified by iTRAQ together with two-dimensional liquid chromatography tandem mass spectrometry (LC-MS/MS). Large-scale identification and different expression analysis of the proteins were performed, and metabolic pathways were constructed, especially active ingredients.

## Results

### Protein profiling and iTRAQ quantification

**Fig. 1:**
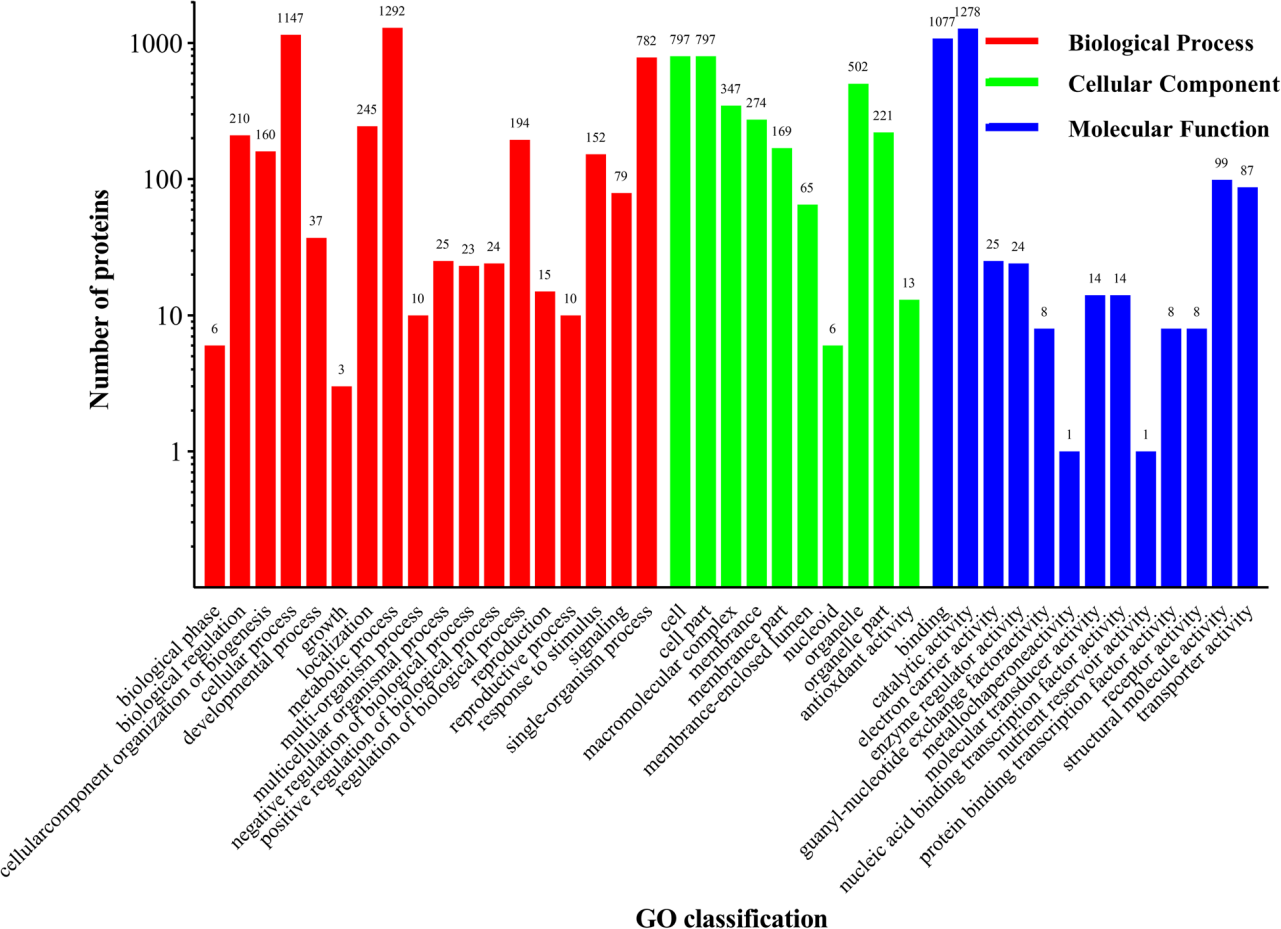
Barplot of the Gene Ontology analysis. The bar chart shows the distribution of corresponding GO terms. Different colors represent different GO categories

**Fig. 2:**
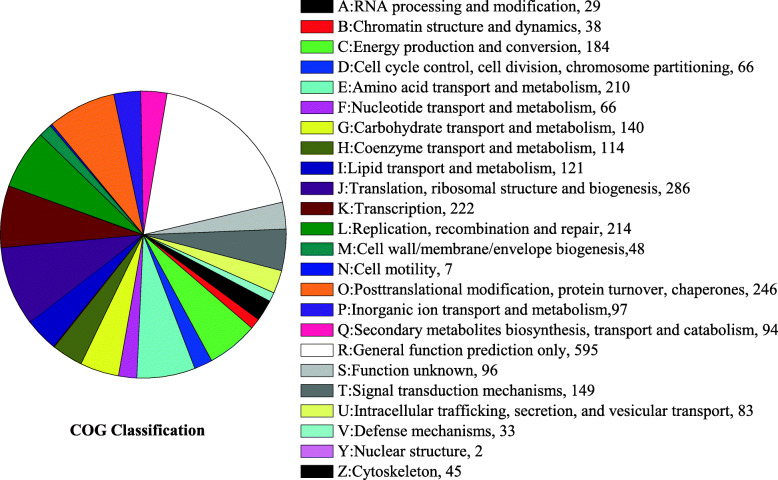
Pie chart of the COG Analysis. All identified proteins are classified into 24 clusters of orthologous groups (COG) categories

In order to obtain the overview of the *O. sinensis* zjut proteome, the protein samples were prepared from the mycelium at different culture periods (3d, 6d and 9d) and relatively quantified by iTRAQ. A total of 371,999 spectra were generated and 4005 proteins included 22,202 peptides were identified (with 1% FDR) (Supplementary information 3: Table S2). GO analysis of total proteins was based on biological process, cellular component and molecular function, and all proteins were classified into 41 functional groups (Fig. 1). Biological processes were associated with the following brief pathways: metabolic process (29.27%), cellular process (25.99%) and single-organism process (17.72%); cellular components were assigned to the following cellular compartments: cell (24.98%), cell part (24.98%) and organelle (15.73%); The most highly enriched molecular functions were binding (48.34%), followed by catalytic activity (40.73%). Moreover, the proteins were further classified into 24 functional categories using COG classifications (Fig. 2). The largest group category was found to have general functions only (18.68%), followed by translation, ribosomal structure and biogenesis (9.00%), and posttranslational modification, protein turnover, chaperones (7.72%). Only a small fraction of the protein was functionally related to the categories of cell motility (0.22%) and nuclear structure (0.06%). About 2.95% of the identified proteins (94 proteins) were related to secondary metabolites biosynthesis, transport and catabolism.

In this study, 6d-VS-3d, 9d-VS-3d, and 9d-VS-6d were set as comparison groups. Corresponding proteins, exhibiting a greater than 1.2-fold change and Q-value less than 0.05, were defined as differentially expressed proteins. Ultimately, 605 DEPs (340 up-regulated proteins and 265 down-regulated proteins) between 6d-VS-3d, 1188 DEPs (545 up-regulated proteins and 643 down-regulated proteins) between 9d-VS-3d, and 428 DEPs (215 up-regulated proteins and 213 down-regulated proteins) between 9d-VS-6d were obtained (Supplementary information 3: Table S3).

### GO and pathway enrichment analysis of DEPs

**Fig. 3:**
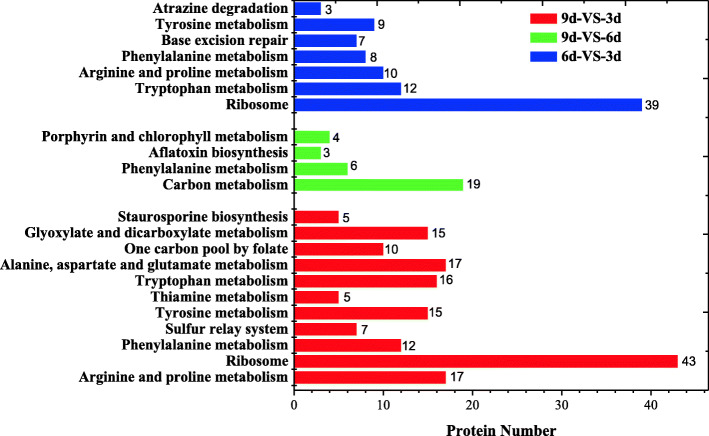
Statistics of pathway enrichment of differentially expressed proteins in the comparison groups of 6d-VS-3d, 9d-VS-3d, and 9d-VS-6d

The GO enrichment analysis of DEPs was shown in Supplementary information 4 (Figure S2, Figure S3, Figure S4). The DEPs were further analyzed by KEGG. As shown in Fig. 3, among the comparison group of 6d-VS-3d, the DEPs were mainly enriched in “ribosome” (39 members), followed by “tryptophan metabolism” (12 members), “arginine and proline metabolism” (10 members), “tyrosine metabolism” (9 members), and “phenylalanine metabolism” (8 members). For the comparison group of 9d-VS-6d, the DEPs were mainly enriched in “carbon metabolism” (19 members) and” phenylalanine metabolism” (6 members). Among the comparison group of 9d-VS-3d, the DEPs were mainly enriched in “ribosome” (43 members), followed by “tyrosine metabolism” (15 members), “phenylalanine metabolism” (12 members), “tryptophan metabolism” (16 members), “arginine and proline metabolism” (17 members), “alanine, aspartate and glutamate metabolism” (17 members), “one carbon pool by folate” (10 members), and “glyoxylate and dicarboxylate metabolism” (15 members).

### Metabolic pathways of D-mannitol, cordycepin and purine nucleotides

**Fig. 4:**
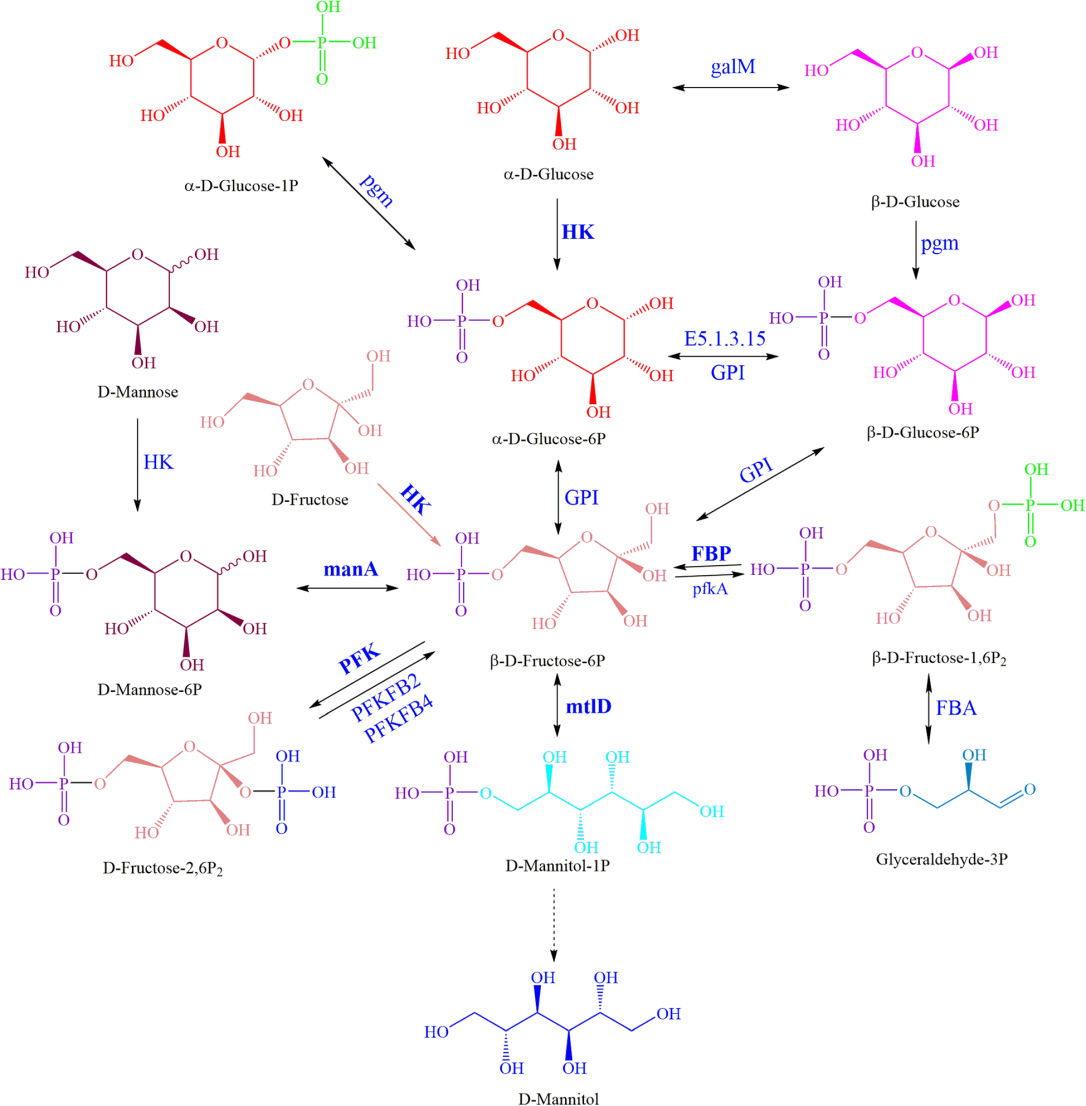
The metabolic pathway of D-mannitol in *O. sinensis* zjut. The biosynthesis pathway of D-mannitol was constructed based on the annotation of the *O. sinensis* zjut proteome. The precursor of D-mannitol is D-mannitol-1P which is formed from β-D-fructose-6P. The dotted line represented the protein did not annotated to the *O. sinensis* zjut proteome. The DEPs (HK, manA, PFK, mtlD, FBP) was shown with bold fonts. HK: Hexokinase; manA: Mannose-6-phosphate isomerase; FBP: Fructose-1,6-bisphosphatase I; pfkA: 6-phosphofructokinase; GPI: Glucose-6-phosphate isomerase; E5.1.3.15: Glucose-6-phosphate 1-epimerase; FBA: Fructose-bisphosphate aldolase; galM: Aldose 1-epimerase; pgm: Phosphoglucomutase; mtlD: Mannitol-1-phosphate 5-dehydrogenase; PFK: 6-phosphofructo-2-kinase; PFKFB2: Fructose-2,6-biphosphatase 2; PFKFB4: Fructose-2,6-biphosphatase 4

**Fig. 5:**
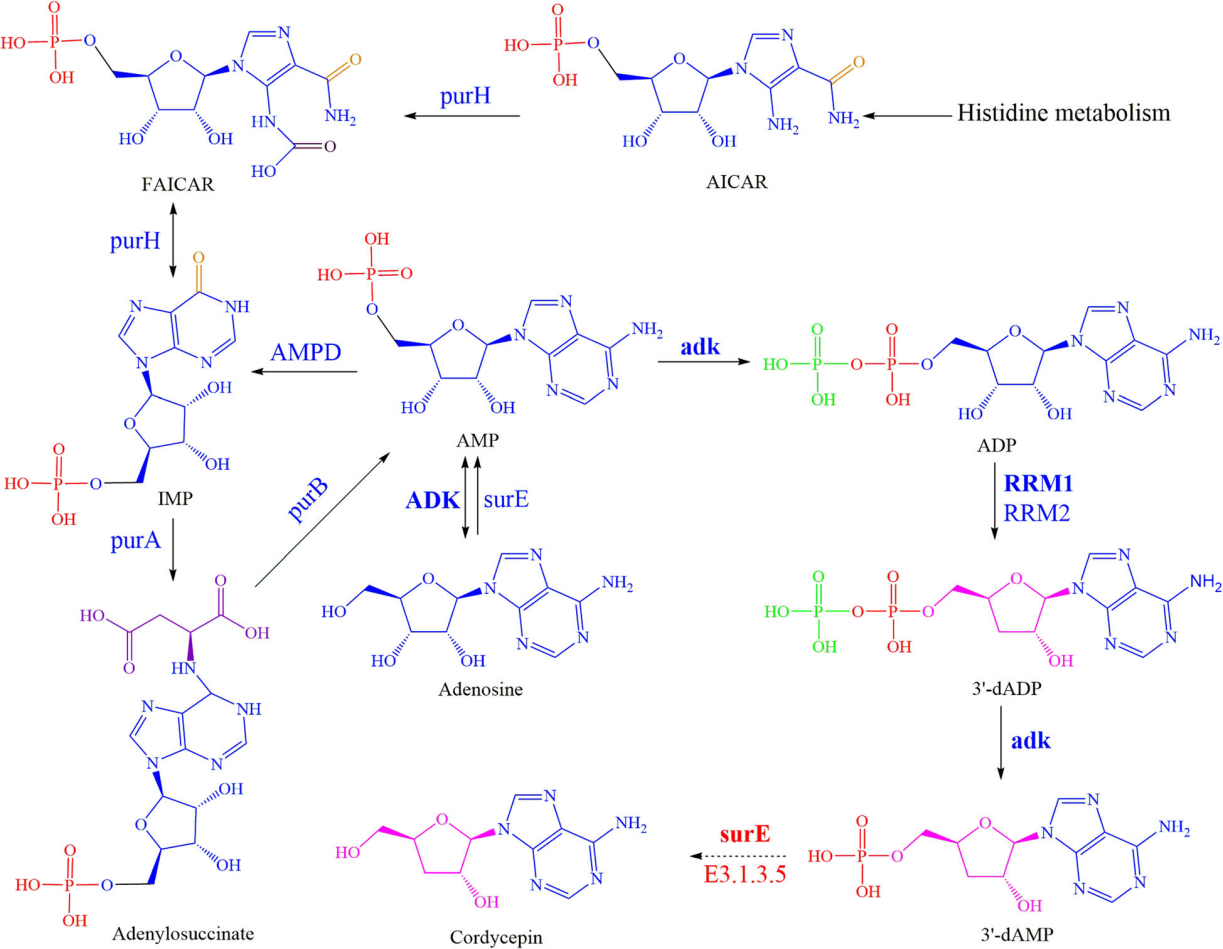
The metabolic pathway of cordycepin in *O. sinensis* zjut. The biosynthesis pathway of cordycepin was constructed based on the annotation of the *O. sinensis* zjut proteome. The dotted line represented the possible protein involved in the conversion of 3′-dAMP to cordycepin. The DEPs (ADK, adk, RRM1, surE) was shown with bold fonts. purH: IMP cyclohydrolase; AMDP: AMP deaminase; adk: Adenylate kinase; purA: Adenylosuccinate synthase; purB: Adenylosuccinate lyase; ADK: Adenosine kinase; surE: 5′-nucleotidase; E3.1.3.5: 5′-nucleotidase; RRM1: Ribonucleoside-diphosphate reductase subunit M1; RRM2: Ribonucleoside-diphosphate reductase subunit M2

Based on the glycolytic pathway and fructose-mannose pathway, the biosynthetic pathway of D-mannitol was successfully constructed (Fig. 4), and the average content of D-mannitol was 10.12% (3rd day 7.16%, 6th day 8.63%, and 9th day 14.58%, Supplementary information 1: Table S1). According to the constructed pathway, the initial precursors of D-mannitol are various saccharide, such as glucose, fructose and mannose. A total of 18 proteins, including 7 DEPs (two up-regulated HK, one up-regulated FBP, one up-regulated mtlD, two down-regulated manA and one down-regulated FBP), were located in the D-mannitol metabolic pathway (Supplementary information 5: Table S4). After several catalytic steps, β-D-fructose-6P is formed, and then converts to D-mannitol-1P which is the immediate precursor of D-mannitol. Unfortunately, we did not find mannitol-1-phosphate phosphatase which catalyzes D-mannitol-1-P to D-mannitol. The biosynthetic pathway of cordycepin, which might originate from histidine and end up with 3′-deoxyadenosine, was constructed (Fig. 5), and the average content of cordycepin was 0.308 mg/g (3rd day 0.327 mg/g, 6th day 0.140 mg/g, and 9th day 0.457 mg/g, Supplementary information 1: Table S1). In the constructed pathway, cordycepin is synthesis following the biosynthesis of adenosine. AMP, the precursor of adenosine, can convert to ADP by the catalysis of adk, and then RRM1 or RRM2 catalyzes ADP to form 3′-dADP. 3′-dADP dephosphorylates and forms 3′-dAMP which may be the immediate precursor of cordycepin. A total of 13 proteins, including 4 DEPs (one up-regulated ADK, one down-regulated adk, one down-regulated surE and one down-regulated RRM1) participated in the biosynthetic pathway of cordycepin (Supplementary information 5: Table S5). In addition, several types of purine nucleosides including inosine, adenosine, guanosine and xanthosine could be synthesized from IMP. The possible metabolic pathways of purine nucleosides and the corresponding proteins were shown in Supplementary information 5 (Figure S5, Table S6).

### Metabolic pathway of monosaccharides

Polysaccharides from *O. sinensis* zjut were made up of mannose, galactose and glucose, and its total content was 3.848% (Supplementary information 1: Table S1). According to the metabolic pathways, there are 18 proteins involved in the biosynthetic pathway of D-mannose (Supplementary information 6: Figure S6, Table S7), 14 proteins involved in D-glucose biosynthesis (Supplementary information 6: Figure S7, Table S8), and 14 proteins involved in D-galactose biosynthesis (Supplementary information 6: Figure S8, Table S9). The metabolic pathways, including corresponding proteins and mRNA involved in these pathways, were shown in Supplementary information 6.

### Amino acid metabolic pathways

Sixteen types of amino acids in *O. sinensis* zjut were detected by amino acid analyzer (Supplementary information 7: Table S10). The amino acid with the highest content in *O. sinensis* zjut was histidine (40.93 mg/g), followed by arginine (36.14 mg/g). The amino acids with the lowest contents was tyrosine (6.23 mg/g), followed by isoleucine (7.25 mg/g), and phenylalanine (8.08 mg/g). The proteins involved in histidine, arginine, phenylalanine and tyrosine biosynthesis were successfully obtained (Supplementary information 8: Table S11, Table S12, Table S13). The synthetic pathways of histidine (Supplementary information 8: Figure S9), arginine (Supplementary information 8: Figure S10), phenylalanine and tyrosine (Supplementary information 8: Figure S11) were successfully constructed, respectively.

### Noteworthy proteins in *O. sinensis* zjut

Based on the analysis of KEGG database, the metabolic pathway of glycolysis/gluconeogenesis and citrate cycle (Supplementary information 10: Figure S12, Figure S13,) were constructed, and the proteins involved in these energy pathways were obtained (Supplementary information 9 and 10: Table S14, Table S15, Table S16). Moreover, 9 proteins (two superoxide dismutase, three catalase, a thioredoxin reductase and three peroxiredoxin) with antioxidant activity were obtained (Supplementary information 11: Table S17), which might play an important role in oxidation resistance.

## Discussion

Recently, the genetic information of *Cordyceps* was gradual enrichment [25, 26], and the productions of active compounds in *H. sinensis* by submerged fermentation were promoted and satisfying on the basis of genetic study [15, 16]. With the studies focusing on genetic information, there were little proteomics reports for artificially-cultivated *O. sinensis.* As we know, gene expression regulation was a complex multilevel process, and the correlation between mRNA and protein abundances was only approximately 27 to 40% [31]. In fact, genes were the carriers of genetic information [32, 33], and proteins were the executors of the physiological functions and direct manifestations of life activity [34]. Therefore, it is urgent and important to enhance proteome analysis of artificially-cultivated *O. sinensis*.

iTRAQ, as a reliable quantitative approach, has widely used in the field of crop proteomics, allowing simultaneous identification and quantification of proteins from multiple samples with high coverage [35–37]. In this study, proteomes of *O. sinensis* zjut at different culture periods were investigated by iTRAQ, and this systematically large-scale study of proteins provided a novel way to study the gene functions and expression difference directly at protein levels in submerged fermentation for *O. sinensis*. Subsequent studies, including function annotation and metabolic pathways construction, were not only the complement and verification to the genetic analysis with genome and transcriptome but also the necessary to fundamental research. As an analytic system, the genome, transcriptome and proteome sequencing analysis could provide a scientific basis for medicinal mechanism to carry out great exploration, application and improvement of submerged fermentation for *O. sinensis*.

Active ingredients of submerged fermentation for *O. sinensis,* such as mannitol, cordycepin and polysaccharide, are used to treat weakness after sickness, lung and kidney-associated diseases, and sexual dysfunction [6, 38, 39]. Recently, the metabolic pathways of active ingredients were constructed at the transcriptional level in *H. sinensis* [26]*.* In this study, the metabolic pathways were also predicted directly at protein level, which played significant roles in further study. For metabolic pathway of mannitol (Fig. 4)*,* mannitol-1-phosphate phosphatase that catalyzes D-mannitol-1-P to D-mannitol was not annotated in proteome, and it was more confidence to the result of the transcriptome study for other phosphatases replaced the function of mannitol-1-phosphate phosphatase or mannitol-1-phosphate phosphatase sequence of *O. sinensis* zjut, which was un-annotated to protein database since its low homology with currently reported mannitol-1-phosphate phosphatases from other organisms [26]. With the proteome analysis of *O. sinensis* zjut*,* the new metabolic pathway of cordycepin (Fig. 5) was different with the conjecture in the transcriptome study. Following the biosynthesis of adenosine, the proteomics result indicated SurE (E3.1.35) (5′-nucleotidase) catalyzes the conversion of 3′-dAMP (3′-adenine deoxynucleotide) to cordycepin rather than N-glycosylation lyase catalyzes the glycosylation exchange between adenosine and cordycepose to generate cordycepin in genetic studies [26, 40]. The constructed metabolic pathways could be the guidance in metabolic engineering study of this organism in future.

Based on the proteomics at different culture periods, DEPs were obtained to be analyzed. As shown in Fig. 4 and Table S4, two up-regulated HKs may boost the conversion of α-D-glucose to α-D-glucose-6P as well as the conversion of D-fructose to β-D-fructose-6P, and the up-regulated FBP may boost the conversion of β-D-fructose-1,6P_2_ to β-D-fructose-6P, leading to the accumulation of β-D-fructose-6P. Two down-regulated manA, one down-regulated PFK and one up-regulated mtlD may convert more β-D-fructose-6P to D-mannit-1P, which might indicate the growth rate of D-mannitol was rise with the culture periods of the *O. sinensis* zjut. There were three down-regulated proteins that might be the key enzymes in the metabolic pathway of cordycepin (Fig. 5 and Table S5). Comparing with the 3rd day, *adk* expression level was down-regulated 0.86-fold on the 6th day and 0.77-fold on the 9th day, *RRM1* expression level was down-regulated 0.87-fold on the 6th day and 0.8-fold on the 9th day, and *surE* expression level was down-regulated 0.65-fold on the 6th day and 0.57-fold on the 9th day. These down-regulated proteins catalyze AMP to ADP, ADP to 3′-dADP, 3′-dADP to 3′-dAMP, 3′-dAMP to cordycepin, respectively. The growth rate of cordycepin was decreased with the culture periods of the *O. sinensis* zjut*,* since adenosine or IMP could be an intermediate for other compounds such as inosine, xanthosine and guanosine [41]. As a secondary metabolic pathway, the low content of cordycepin on 6th day (3rd day 0.327 mg/g, 6th day 0.140 mg/g, and 9th day 0.457 mg/g) might be caused by periodical release. The DEPs involved in mannitol and cordycepin biosynthesis play important functions, and studies focus on these enzymes would pave a theoretical foundation to regulate and promote the active ingredients productions of submerged fermentation by *O. sinensis*.

Previous study reported that sixteen to eighteen types of amino acids existed in *O. sinensis* [42]. In this study, sixteen types of amino acids were obtained in *O. sinensis* zjut including 7 essential amino acids and 13 pharmaceutical amino acids. As shown in Supplementary information 7: Table S10, the contents of most individual amino acids were discrepant between *O. sinensis* zjut and natural *O. sinensis*. The two principal amino acids and their levels in *O. sinensis* zjut were 13.36% histidine and 11.80% arginine, compared with 3.08% histidine and 5.54% arginine in natural *O. sinensis.* Furthermore, the content of valine, which was the most different amino acid, was 7.98% in *O. sinensis* zjut*,* compared with 1.03% in *O. sinensis*. The variation in *O. sinensis* zjut and natural *O. sinensis* might be associated with geography, climate and nutrition differences.

In this study, we found some noteworthy pathways and proteins in *O. sinensis* zjut. Fatty acids, which might protect organism from cold, are the major energy sources for *O. sinensis* zjut [43, 44]. As the major sources for metabolic intermediates [45, 46], two energy pathways (glycolysis/gluconeogenesis pathway and the citrate cycle) might also provide energy for *O. sinensis* zjut and protect it from cold. Recently, superoxide dismutase was extracted from *O. sinensis* [47], to study for powerful anti-inflammatory and pharmacological activities [30, 48]. Two superoxide dismutases, which were responsible for converting superoxide to O_2_ and H_2_O_2_, were found in *O. sinensis* zjut. Among the proteinases, some special functional proteins have been found, including two secreted aspartic proteinases, which are important hydrolases and potent antifungal agents [49, 50]. Moreover, the proteins with special function could be cloned and then expressed in *Escherichia coli* to study the enzymatic property for exploring its industry application [51, 52].

## Conclusion

In this study, iTRAQ technology together with two-dimensional liquid chromatography tandem mass spectrometry were first used to relatively quantify the proteins of *O. sinensis* zjut at different culture periods. In total, 4005 proteins were obtained and further annotated, and the metabolic pathways of D-mannitol, cordycepin, monosaccharides, two high content (histidine and arginine) and two low content amino acids (phenylalanine and tyrosine), and fatty acid were constructed at the protein level. The important proteins involved in metabolic pathways of active ingredients were found by comparative proteomics of *O. sinensis* zjut at different culture periods. In addition, two energy pathways (glycolysis/gluconeogenesis pathway and the citrate cycle) were described. Proteins with antioxidant and antifungal activity were also obtained, which could be cloned, expressed and studied in the future. This work provided scientific basis to study protein profile and comparison of protein expression levels of submerged fermentation for *O. sinensis*, and it will be helpful for metabolic engineering works to active ingredients for exploration, application and improvement of this strain.

## Methods

### Strains, medium and culture conditions

Wild *O. sinensis* samples were collected from the slopes of 4000–4300 m above sea level on the Qinghai-Tibet plateau in Yushu city, Qinghai Province. The isolation and identification processes as well as preservation information of *O. sinensis* zjut were referred to a previous study [26]. The medium (pH 7.0) for *O. sinensis* zjut cultivation contained glucose (1.5%), corn powder (1%), silkworm chrysalis meal (2%), MgSO_4_ (0.05%), and KH_2_PO_4_ (0.05%). The strain was grown on the medium using 200 L submerged stirred fermenter at 16 °C. The single colonies, growth curve and active compounds contents of *O. sinensis* zjut were performed (Supplementary information 1: Figure S1, Table S1). The samples for proteome analysis were harvested on the 3rd, 6th and 9th days.

### iTRAQ quantitative proteome

The quantitative proteome was carried out by iTRAQ according to the standard experimental protocol. The details of the iTRAQ procedure were shown in Supplementary information 2.

### Protein quantification

The software Iquant was used for quantitatively analyzing the labeled peptides with isobaric tags [53]. To assess the confidence of peptides, the PSMs (propensity matching analysis) were pre-filtered at a PSM-level FDR of 1%. Identified peptide sequences were assembled into a set of confident proteins with the parsimony principle.

### Function analysis

Enrichment analysis of the DEPs was conducted according to the information from Kyoto Encyclopedia of Genes and Genome (KEGG) pathway and Gene Ontology (GO) databases, respectively, using the following formula:

$$ \mathrm{P}=1-\sum \limits_{i=0}^{m-1}\frac{\left(\underset{i}{M}\right)\left(\underset{n-i}{N-M}\right)}{\left(\underset{n}{N}\right)} $$

N is the number of all proteins that can be annotated to GO or KEGG, n is the number of DEPs in N, M is the number of proteins that can be annotated to a certain GO term or KEGG pathway, and m is the number of DEPs in M.

Blast2GO tool (23) was used to categorize the identified proteins according to their functions, biological process and cellular component. The metabolic pathway analysis was conducted according to the KEGG Pathway Database (http://www.genome.jp/kegg/pathway.html).

### Statistical analysis

Unless otherwise noted, all the experiments in this study were performed in triplicate. An analysis of variance (ANOVA) was performed using the SAS program version 8.1 (SAS Institute Inc., Cary, NC, USA). The least significant difference (LSD) was computed at *p* < 0.05. All the figures in this study were processed using the Origin software version 8.0 (OriginLab Corp., Northampton, MA, USA).

### Availability of data and materials

The mass spectrometry proteomics data have been deposited to the ProteomeXchange Consortium (http://proteomecentral.proteomexchange.org/) via the iProX partner repository [54] with the dataset identifier PXD016899.

## Supplementary Information

**Additional file 1: Supporting information 1**: The single colonies, growth curve and active compounds contents in *O. sinensis* zjut; **Supporting information 2** The details of iTRAQ quantitative proteomics; **Supporting information 3**: The identified results of the samples and DEPs in *O. sinensis* zjut; **Supporting information 4**: GO enrichment analysis of DEPs in *O. sinensis* zjut; **Supporting information 5**: The proteins involved in metabolic pathway of D-mannital, cordycepin and purine nucleotides in *O. sinensis* zjut, and purine nucleotides metabolic pathway; **Supporting information 6**: The metabolic pathways of D-mannose, D-galactose and D-glucose as well as the proteins involved in these pathways; **Supporting information 7**: The contents of sixteen types of amino acids in *O. sinensis* zjut; **Supporting information 8**: The metabolic pathways of the histidine, arginine, phenylalanine and tyrosine as well as the proteins involved in these pathways; **Supporting information 9**: The proteins involved in the biosynthetic pathway of fatty acids; **Supporting information 10**: The metabolic pathways of glycolysis/gluconeogenesis and citrate cycle as well as the proteins involved in these pathways; **Supporting information 11**: The proteins with anti-oxidant activity in *O. sinensis* zjut.

## Data Availability

All data generated or analyzed during this study are included in this article and its supplementary information files.

## Abbreviations

iTRAQ: Isobaric tags for relative and absolute quantification
FDR: False discovery rate
DEPs: Differentially expressed proteins
PSMs: Propensity matching analysis
