# Supplementary material for: Proteome sequencing and analysis of Ophiocordyceps sinensis at different culture periods
Source: BMC Genomics. 2020 Dec 11;21:886. doi: 10.1186/s12864-020-07298-z (PMC7731760; doi:10.1186/s12864-020-07298-z)
Supplement: Supplementary file 1 — Additional file 1: Supporting information 1: The single colonies, growth curve and active compounds contents in O. sinensis zjut; Supporting information 2 The details of iTRAQ quantitative proteomics; Supporting information 3: The identified results of the samples and DEPs in O. sinensis zjut; Supporting information 4: GO enrichment analysis of DEPs in O. sinensis zjut; Supporting information 5: The proteins involved in metabolic pathway of D-mannital, cordycepin and purine nucleotides in O. sinensis zjut, and purine nucleotides metabolic pathway; Supporting information 6: The metabolic pathways of D-mannose, D-galactose and D-glucose as well as the proteins involved in these pathways; Supporting information 7: The contents of sixteen types of amino acids in O. sinensis zjut; Supporting information 8: The metabolic pathways of the histidine, arginine, phenylalanine and tyrosine as well as the proteins involved in these pathways; Supporting information 9: The proteins involved in the biosynthetic pathway of fatty acids; Supporting information 10: The metabolic pathways of glycolysis/gluconeogenesis and citrate cycle as well as the proteins involved in these pathways; Supporting information 11: The proteins with anti-oxidant activity in O. sinensis zjut. [file 12864_2020_7298_MOESM1_ESM.doc]

**Proteome sequencing and analysis of** ***Ophiocordyceps sinensis* at different culture periods**

## Authors

Bo Zhang^1^, Bo Li^1^, Xiao-Hui Men^1^, Zhe-Wen Xu^1^, Hui Wu^2, 3^, Xiang-Tian Qin^2, 3^, Feng Xu^2, 3^, Yi Teng^2, 3^, Shui-Jin Yuan^2, 3^, Li-Qun Jin^1^, Zhi-Qiang Liu^1^*, Yu-Guo Zheng^1^

^1^Key Laboratory of Bioorganic Synthesis of Zhejiang Province, College of Biotechnology and Bioengineering, Zhejiang University of Technology, Hangzhou 310014, China

^2^HuaDong Medicine (Hangzhou) Bailing Biological Technology Co., Ltd, Hangzhou 311220, China.

^3^East China Pharmaceutical Group Limited Co., Ltd, Hangzhou 311000, China.

*Corresponding author:

Tel: +86-571-88320614, Fax: +86-571-88320630, E-mail: microliu@zjut.edu.cn

**Table of Contents**

Supporting information 1: The single colonies, growth curve and active compounds contents in *O. sinensis* zjut*.*

Supporting information 2: The details of iTRAQ quantitative proteomics.

Supporting information 3: The identified results of the samples and DEPs in *O. sinensis* zjut*.*

Supporting information 4: GO enrichment analysis of DEPs in *O. sinensis* zjut.

Supporting information 5: The proteins involved in metabolic pathway of D-mannitol, cordycepin and purine nucleotides in *O. sinensis* zjut, and purine nucleotides metabolic pathway.

Supporting information 6: The metabolic pathways of D-mannose, D-galactose and D-glucose as well as the proteins involved in these pathways.

Supporting information 7: The contents of sixteen types of amino acids in *O. sinensis* zjut*.*

Supporting information 8: The metabolic pathways of the histidine, arginine, phenylalanine and tyrosine as well as the proteins involved in these pathways.

Supporting information 9: The proteins involved in the biosynthetic pathway of fatty acids.

Supporting information 10: The metabolic pathways of glycolysis/gluconeogenesis and citrate cycle as well as the proteins involved in these pathways.

Supporting information 11: The proteins with anti-oxidant activity in *O. sinensis* zjut*.*

**Supporting information 1: The single colonies, growth curve and active compounds contents in** ***O. sinensis* zjut*.***

A

B


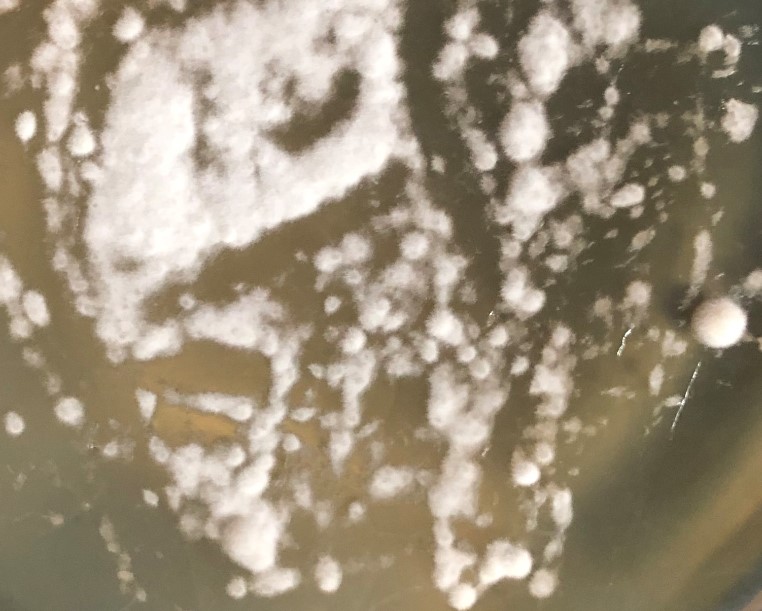


**Figure. S1 The colonial morphology and growth curve of *O. sinensis* zjut.** A: The colonial morphology of *O. sinensis* zjut is irregular, and the color of single colonies was white with the diameter ranged from 1 cm to 2 cm. Hyphae of *O. sinensis* zjut were fluffy and outward. B: The growth curve of *O. sinensis* zjut.

**Table S1. The contents of active compounds in** ***O. sinensis* zjut*.***

| **Active Compound** | ***O. sinensis* zjut mycelium** |
| --- | --- |
| D-mannitol | 10.13 % |
| Cordycepin | 0.308 mg/g |
| Uridine | 3.92 mg/g |
| Vernine | 0.181 mg/g |
| Adenosine | 1.35 mg/g |
| Thymidine | 0.55 mg/g |
| Total polysaccharide | 3.848 % |
| Fatty acid | 5.66 % |

**Supporting information 2: The details of iTRAQ quantitative proteomics.**

**Protein Preparation**

6 Samples were extracted with Lysis buffer 3 (8 M Urea, 40 mM Tris-HCl or TEAB, pH 8.5) containing 1 mM phenylmethanesulfonyl fluoride (PMSF) and 2 mM Ethylene Diamine Tetraacetic Acid (EDTA final concentration). Placing 6 samples on ice for 5 min, 10 mM DTT (final concentration) was added to each sample. The suspension was sonicated at 200 W for 1 min and then centrifuged at 4 °C, 25000g for 20 min. The supernatant was incubated at 56 °C for 1 h. Subsequently, after cooling to room temperature, the sample was incubated with 55 mM IAM (final concentration) for 45 min in the dark room for alkylation. The supernatant containing proteins was quantified by Bradford after centrifuge with 25000g×20 min at 4 °C.

**QC of Protein Extraction**

**(1) Protein Quantitation with Bradford assay**

Add 0, 2, 4, 6, 8, 10, 12, 14, 16 and 18 μl BSA (0.2 μg/μl) solution separately to a 96-well plate, and then add 20, 18, 16, 14, 12, 10, 8, 6, 4 and 2 μl pure water to the corresponding wells. Also prepare serial dilutions (20 μl each well) of the unknown sample to be measured. Add 180 μl of Coomassie Blue to each well and mix. Read the absorbance of each standard and sample well at 595 nm. Each sample has at least two duplicates. Plot the absorbance of the standards vs. their concentration. Compute the extinction coefficient and calculate the concentrations of the unknown samples.

**(2) SDS-PAGE**

Mix 15-30 μg proteins with loading buffer in centrifuge tube and heat them at 95℃ for 5 minutes. Then, centrifuge it at 25000×g for 5 minutes and load the supernatant to sample holes in 12% polyacrylamide gel. Run SDS-PAGE in constant voltage at 120V for 120 minutes. Once it was finished, stain gel with Coomassie Blue for 2 hours, then add destaining solution (40% ethanol and 10% acetic acid) and put it on a shaker (exchange destaining solution for 3~5times, 30 minutes a time).

**Protein Digestion and Peptide Labeling**

The solution of protein solution (100 ug) with 8M urea was diluted 4 times by 100 mM TEAB. Trypsin Gold (Promega, Madison, WI, USA) was used to digest the proteins with the ratio of protein : trypsin = 40:1 at 37 °C overnight. After trypsin digestion, peptides were desalted with a Strata X C18 column (Phenomenex) and vacuum-dried according to the manufacturer's protocol. The peptides were dissolved in 30 ul 0.5 M TEAB with vortexing. After the iTRAQ labeling reagents were recovered to ambient temperature, they were transferred and combined with proper samples. Peptide labeling was performed by iTRAQ Reagent 8-plex Kit according to the manufacturer's protocol. The labeled peptides with different reagents were combined and desalted with a Strata XC18 column (Phenomenex) and vacuum-dried according to the manufacturers protocol.

**Peptide Fractionation with Strong Cation Exchange Chromatography**

The peptides were separated on a Shimadzu LC-20AB HPLC Pump system coupled with a high pH RP column and then reconstituted with buffer A (5% ACN, 95% H_2_O, adjust pH to 9.8 with ammonia) to 2 ml and loaded onto a column containing 5 μm particles (Phenomenex). The peptides are separated at a flow rate of 1 mL/min with a gradient of 5% buffer B (5% H_2_O, 95% ACN, adjust pH to 9.8 with ammonia) for 10 min, 5-35% buffer B for 40 min, 35-95% buffer B for 1 min. Then, the system is maintained in 95% buffer B for 3 min and decreases to 5% in less than 1 min before equilibrating with 5% buffer B for 10 min. Elutionis were measured at the absorbance of 214 nm monitoredby, and fractions are collected every 1 min. The eluted peptides are devided into 20 fractions and vacuum-dried. And then, each fraction was resuspended in buffer A (2% ACN and 0.1% FA in water) and centrifuged at 20000×g for 10 min. Loading the supernatant onto a C 18 trapcolumn 5 μL/min for 8 min using a LC-20AD nano-HPLC instrument (Shimadzu, Kyoto, Japan) by the auto sampler. Then, the peptides were eluted from trap column and separated by an analytical C18 column (inner diameter 75 μm) packed in-house. The gradient was run at 300 mL/min starting from 8 to 35% of buffer B (2% H_2_O and 0.1% FA in ACN) in 35 min, then up to 60% in 5 min, then maintenance at 80% B for 5 minutes, and finally return to 5% in 0.1 min and equilibrated for 10 min.

**LC-MS/MS Analysis**

Data acquisition was performed with a Triple TOF 5600 System (SCIEX, Framingham, MA, USA) which was equipped with a Nanospray III source (SCIEX, Framingham, MA, USA), a pulled quartz tip as the emitter (New Objectives, Woburn, MA) and controlled by software Analyst 1.6 (AB SCIEX, Concord, ON). Data was obtained under the following MS conditions: ion spray voltage 2300 V, curtain gas of 30, nebulizer gas of 15, and interface heater temperature of 150 °C. High sensitivity mode was used for the whole data acquisition. The accumulation time for MS1 is 250 ms, and the mass was ranged from 350 to 1500 Da. Based on the intensity in MS1 survey, as many as 30 product ion scans were collected if exceeding a threshold of 120 counts per second (counts/s) and with charge-state 2+ to 5+, dynamic exclusion was set for 1/2 of peak width (12 s). For iTRAQ data acquisition, the collision energy was adjusted to all precursor ions for collision-induced dissociation and the Q2 transmission window for 100Da was 100%.

**Supporting information 3: The identified results of the samples and DEPs in *O. sinensis* zjut*.***

***Table S2. The identified results of the samples in O. sinensis zjut.***

| **Sample name** | **Total spectra** | **Spectra** | **Unique Spetra** | **Peptide** | **Unique Peptide** | **Protein** |
| --- | --- | --- | --- | --- | --- | --- |
| *O. sinensis* zjut | 371999 | 78386 | 77378 | 22202 | 21963 | 4005 |

***Table S3. The DEPs in O. sinensis zjut.***

| **Comparison_group/**  **protein number** | **Up-regulated** | **Down-regulated** | **All-regulated** |
| --- | --- | --- | --- |
| 6d-VS-3d | 340 | 265 | 605 |
| 9d-VS-3d | 545 | 643 | 1,188 |
| 9d-VS-6d | 215 | 213 | 428 |

**Supporting information 4: GO enrichment analysis of DEPs** **in *O. sinensis* zjut*.***

**GO enrichment analysis of DEPs in the comparison group of 6d-VS-3d**

Among the comparison group of 6d-VS-3d (605 DEPs) (Fig. S2), the DEPs were mainly enriched in “non-membrane-bounded organelle” (46 members), “intracellular non-membrane-bounded organelle” (46 members), “cytoplasmic part” (68 members), “macromolecular complex” (69 members), “structural constituent of ribosome” (34 members), “structural molecule activity” (34 members), “translation” (43 members), “gene expression” (61 members), “macromolecule biosynthetic process” (51 members), “cellular protein metabolic process” (60 members), “cellular macromolecule biosynthetic process” (50 members) and “cellular macromolecule metabolic process” (89 members).

**Figure. S2 Barplot of the 6d-VS-3d GO Enrichment Analysis.** The bar chart shows the distribution of corresponding enriched GO terms. Different colors represent different GO categories.

**GO enrichment analysis of DEPs in the comparison group of 9d-VS-6d**

Among the comparison group of 9d-VS-6d (Fig. S3), the mainly enriched terms of Cellullar Component were “ribonucleoprotein complex” (24 members), “ribosome” (18 members), “intracellular non-membrane-bounded organelle” (26 members), “non-membrane-bounded organelle” (26 members), “organelle” (56 members), “intracellular organelle” (56 members), “macromolecular complex” (36 members), “intracellular part” (71 members), “cytoplasmic part” (33 members)， and “nucleus” (20 members).


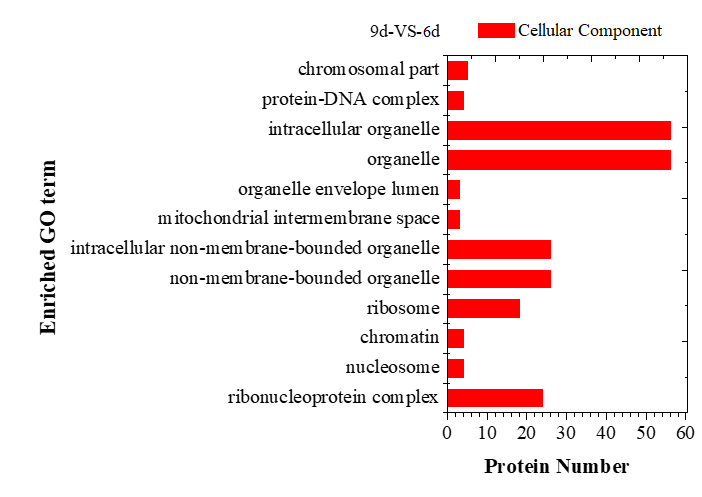


**Figure. S3 Barplot of the 9d-VS-6d GO Enrichment Analysis.** The bar chart shows the distribution of corresponding enriched GO terms. Different colors represent different GO categories.

**GO enrichment analysis of DEPs in the comparison group of 9d-VS-3d**

Among the comparison group of 9d-VS-3d (1188 DEPs) (Fig. S4), the DEPs were mainly enriched in “ribonucleoprotein complex” (60 members), “ribosome” (46 members), “non-membrane-bounded organelle” (64 members), “intracellular non-membrane-bounded organelle” (64 members), “structural constituent of ribosome” (36 members), and “structural molecule activity” (39 members).

**Figure. S4 Barplot of the 9d-VS-3d GO Enrichment Analysis.** The bar chart shows the distribution of corresponding enriched GO terms. Different colors represent different GO categories.

**Supporting information 5: The proteins involved in metabolic pathway of D-mannitol, cordycepin and purine nucleotides in *O. sinensis* zjut, and purine nucleotides metabolic pathway.**

**Table. S4 The proteins involved in D-mannitol metabolic pathway.**

| **Name** | **Definition** | **Protein ID** | **Ratio of 6d-VS-3d** | **Ratio of 9d-VS-3d** | **Corresponding mRNA ID** |
| --- | --- | --- | --- | --- | --- |
| HK | hexokinase | Hirsutella_00142 Hirsutella_02360 Hirsutella_02164 Hirsutella_01203 | -  -  1.22  1.38 | -  -  1.69  1.30 | Hirsutella_08364  Hirsutella_0014  Hirsutella_02360  Hirsutella_02164  Hirsutella_01203  Hirsutella_00666 |
| manA | mannose-6-phosphate isomerase | Hirsutella_03584 Hirsutella_05648 | 0.37  0.80 | 0.51  0.70 | Hirsutella_03584  Hirsutella_07256  Hirsutella_05648 |
| FBP | fructose-1,6-bisphosphatase I | Hirsutella_04940 | 1.2 | 1.51 | Hirsutella_04940 |
| pfkA | 6-phosphofructokinase | Hirsutella_00059 | - | - | Hirsutella_00059 |
| GPI | glucose-6-phosphate isomerase | Hirsutella_05745 Hirsutella_08675 | -  - | -  - | Hirsutella_05745  Hirsutella_08675 |
| E5.1.3.15 | glucose-6-phosphate 1-epimerase | Hirsutella_08699 | - | - | Hirsutella_08308  Hirsutella_07356  Hirsutella_08699 |
| FBA | fructose-bisphosphate aldolase | Hirsutella_07507 | - | - | Hirsutella_07507  Hirsutella_01064 |
| galM | aldose 1-epimerase | Hirsutella_03840 | - | - | Hirsutella_03840  Hirsutella_06104 |
| pgm | phosphoglucomutase | Hirsutella_05031 | - | - | Hirsutella_05031 |
| mtlD | mannitol-1-phosphate 5-dehydrogenase | Hirsutella_05722 | 1.41 | 1.61 | Hirsutella_05722 |
| PFK | 6-phosphofructo-2-kinase | Hirsutella_05862 | 0.78 | 0.74 | Hirsutella_05862 |
| PFKFB2 | fructose-2,6-biphosphatase 2 | Hirsutella_04241 Hirsutella_04245 | -  - | -  - | Hirsutella_04243  Hirsutella_04245  Hirsutella_04241  Hirsutella_01142 |
| PFKFB4 | fructose-2,6-biphosphatase 4 | Hirsutella_05911 | - | - | Hirsutella_05911 |

*NOTE*: The ratio of 6d-VS-3d and 9d-VS-3d means the relative expression amount of protein at different time. >1 means up-regulated expression, <1 means down-regulated expression, “-” means no change.

***Table. S5 The proteins involved in cordycepin metabolic pathway.***

| **Name** | **Definition** | **Protein ID** | **Ratio of 6d-VS-3d** | **Ratio of 9d-VS-3d** | **Corresponding mRNA ID** |
| --- | --- | --- | --- | --- | --- |
| purH | IMP cyclohydrolase | Hirsutella_02072 Hirsutella_07249 | -  - | -  - | Hirsutella_02072 Hirsutella_07249 |
| AMDP | AMP deaminase | Hirsutella_06501 | - | - | Hirsutella_06501 Hirsutella_06297 |
| adk | adenylate kinase | Hirsutella_01431 | 0.86 | 0.77 | Hirsutella_07346 Hirsutella_01431 |
| purA | adenylosuccinate synthase | Hirsutella_05672  Hirsutella_02302 | -  - | -  - | Hirsutella_05672  Hirsutella_02302 |
| purB | adenylosuccinate lyase | Hirsutella_03939 | - | - | Hirsutella_03939 |
| ADK | adenosine kinase | Hirsutella_03704 | 1.19 | 1.52 | Hirsutella_03704 |
| surE | 5'-nucleotidase | Hirsutella_07305 Hirsutella_04882 | 0.65  - | 0.57  - | Hirsutella_07305 Hirsutella_04882 |
| E3.1.3.5 | 5'-nucleotidase | Hirsutella_06211 | - | - | Hirsutella_06211 |
| RRM1 | ribonucleoside-diphosphate reductase subunit M1 | Hirsutella_06344 | 0.87 | 0.8 | Hirsutella_06344 |
| RRM2 | ribonucleoside-diphosphate reductase subunit M2 | Hirsutella_09743 | - | - | Hirsutella_09743 |

*NOTE*: The ratio of 6d-VS-3d and 9d-VS-3d means the relative expression amount of protein at different time. >1 means up-regulated expression, <1 means down-regulated expression, “-” means no change.

**Figure. S5 Purine nucleosides metabolic pathway in *O. sinensis* zjut*.*** Blue part stands for proteins involved in this pathway, and red part stands for protein did not find.

**Table. S6 The proteins involved in purine nucleosides metabolic pathway**

| **Name** | **Definition** | **Protein ID** | **Corresponding mRNA ID** |
| --- | --- | --- | --- |
| AMDP | AMP deaminase | Hirsutella_06501 | Hirsutella_06501 Hirsutella_06297 |
| adk | adenylate kinase | Hirsutella_01431 | Hirsutella_07346 Hirsutella_01431 |
| purA | adenylosuccinate synthase | Hirsutella_05672  Hirsutella_02302 | Hirsutella_05672  Hirsutella_02302 |
| purB | adenylosuccinate lyase | Hirsutella_03939 | Hirsutella_03939 |
| E2.7.1.20 | adenosine kinase | Hirsutella_03704 | Hirsutella_03704 |
| surE | 5'-nucleotidase | Hirsutella_07305 Hirsutella_0488 | Hirsutella_07305  Hirsutella_0488 |
| E3.1.3.5 | 5'-nucleotidase | Hirsutella_06211 | Hirsutella_06211 |
| IMPDH | IMP dehydrogenase | Hirsutella_05563 | Hirsutella_05563 |
| guaA | GMP synthase | Hirsutella_00584 Hirsutella_01517 | Hirsutella_00584 Hirsutella_01517 Hirsutella_08800 |
| APRT | adenine phosphoribosyltransferase | - | Hirsutella_00224 |
| E3.5.4.3 | guanine deaminase | Hirsutella_09346 | Hirsutella_09346 Hirsutella_05663 Hirsutella_08059 Hirsutella_07476 |
| add | adenosine deaminase | Hirsutella_01709 | Hirsutella_01709 |
| CECR1 | adenosine deaminase CECR1 | Hirsutella_08191 | Hirsutella_06370 Hirsutella_08191 |

*NOTE:* “-” means no corresponding protein.

**Supporting information 6: The metabolic pathways of D-mannose, D-galactose and D-glucose as well as the proteins involved in these pathways.**

**Figure. S6 The metabolic pathway of D-mannose.** Blue part stands for proteins involved in this pathway

**Table. S7 The proteins involved in D-mannose metabolic pathway**

| **Name** | **Definition** | **Protein ID** | **Corresponding mRNA ID** |
| --- | --- | --- | --- |
| HK | hexokinase | Hirsutella00142 Hirsutella02360 Hirsutella02164 Hirsutella01203 | Hirsutella_08364  Hirsutella_00142  Hirsutella_02360  Hirsutella_00666  Hirsutella_02164  Hirsutella_01203 |
| manA | mannose-6-phosphate isomerase | Hirsutella03584 Hirsutella05648 | Hirsutella_03584  Hirsutella_07256  Hirsutella_05648 |
| FBP | fructose-1,6-bisphosphatase I | Hirsutella04940 | Hirsutella_04940 |
| pfkA | 6-phosphofructokinase | Hirsutella00059 | Hirsutella_00059 |
| GPI | glucose-6-phosphate isomerase | Hirsutella05745 Hirsutella08675 | Hirsutella_05745  Hirsutella_08675 |
| E5.1.3.15 | glucose-6-phosphate 1-epimerase | Hirsutella08699 | Hirsutella_08308  Hirsutella_07356  Hirsutella_08699 |
| FBA | fructose-bisphosphate aldolase | Hirsutella07507 | Hirsutella_07507  Hirsutella_01064 |
| galM | aldose 1-epimerase | Hirsutella03840 | Hirsutella_03840  Hirsutella_06104 |
| pgm | phosphoglucomutase | Hirsutella05031 | Hirsutella_05031 |
| PFK | 6-phosphofructo-2-kinase | Hirsutella05862 | Hirsutella_05862 |
| PFKFB2 | fructose-2,6-biphosphatase 2 | Hirsutella04241 Hirsutella04245 | Hirsutella_04243  Hirsutella_04245  Hirsutella_04241  Hirsutella_0114 |
| PFKFB4 | fructose-2,6-biphosphatase 4 | Hirsutella05911 | Hirsutella_05911 |

**Figure. S7 The metabolic pathway of D-Glucose.** Blue part stands for proteins involved in this pathway

**Table. S8 The proteins involved in the D-Glucose metabolic pathway**

| **Name** | **Definition** | **Protein ID** | **Corresponding mRNA ID** |
| --- | --- | --- | --- |
| HK | hexokinase | Hirsutella_02360  Hirsutella_02164  Hirsutella_01203  Hirsutella_00142 | Hirsutella_08364  Hirsutella_00142  Hirsutella_02360  Hirsutella_00666  Hirsutella_02164  Hirsutella_01203 |
| GPI | glucose-6-phosphate isomerase | Hirsutella_05745 Hirsutella_08675 | Hirsutella_05745 Hirsutella_08675 |
| pgm | phosphoglucomutase | Hirsutella_05031 | Hirsutella_05031 |
| UGP2 | UTP--glucose-1-phosphate uridylyltransferase | Hirsutella_02382 | Hirsutella_02382 |
| TPS | trehalose 6-phosphate synthase | Hirsutella_03650 Hirsutella_02821 | Hirsutella_04502  Hirsutella_02821  Hirsutella_03650  Hirsutella_07089 |
| otsA | trehalose 6-phosphate synthase | Hirsutella_0431 | Hirsutella_0431 |
| TREH | alpha-trehalase | Hirsutella_03581 | Hirsutella_03581  Hirsutella_06554  Hirsutella_09447 |
| E2.4.1.34 | 1,3-beta-glucan synthase | Hirsutella_03323 Hirsutella_00474 | Hirsutella_00474  Hirsutella_07178  Hirsutella_03323  Hirsutella_02764 |
| EGLC | Glucanendo-1,3-beta-D-glucosidase | Hirsutella_07438 Hirsutella_01170 | Hirsutella_07438  Hirsutella_01170 |

**Figure. S8 The metabolic pathway of D-Galactose.** Blue part stands for proteins involved in this pathway

**Table. S9 The proteins involved in D- Galactose metabolic pathway.**

| **Name** | **Definition** | **Protein ID** | **Corresponding mRNA ID** |
| --- | --- | --- | --- |
| UGP2 | UTP--glucose-1-phosphate uridylyltransferase | Hirsutella_02382 | Hirsutella_02382 |
| galT | UDPglucose--hexose-1-phosphate uridylyltransferase | Hirsutella_01722 | Hirsutella_01722 |
| lacZ | beta-galactosidase | Hirsutella_0620 | Hirsutella_00164  Hirsutella_06201  Hirsutella_07802 |

**Supporting information 7: The contents of sixteen types of amino acids in *O. sinensis* zjut*.***

***Table. S10 The contents of amino acids in O. sinensis zjut and O. sinensis***

| **Amino acid Composition** | **Mycelium of *O. sinensis* zjut（mg/g）** | **Fermentation filtrate of *O. sinensis* zjut (mg/mL)** | **Natural** ***O sinensis*（mg/g）** |
| --- | --- | --- | --- |
| Aspartic acid | 24.430 | 0.128 | 19.3 |
| Threonine | 13.875 | n.d. | 14.8 |
| Glutamic acid | 14.450 | 0.052 | 13.7 |
| Glycine | 28.250 | 0.172 | 35.6 |
| Alanine | 15.850 | 0.344 | 13.6 |
| Cystenine | 24.500 | 0.226 | 23.0 |
| Valine | 24.430 | n.d. | 3.0 |
| Methionine | 14.875 | n.d. | 17.2 |
| Lysine | 9.750 | n.d. | 7.8 |
| Isoleucine | 7.250 | n.d. | 12.2 |
| Leucine | 17.550 | 0.031 | 10.5 |
| Tyrosine | 6.230 | n.d. | 32.4 |
| Phenylalanine | 8.080 | n.d. | 14.2 |
| Histidine | 40.930 | 0.186 | 9.0 |
| Lysine | 19.700 | 0.118 | 26.9 |
| Arginine | 36.130 | n.d. | 16.2 |
| Total amino acid | 306.280 | 2.530 | 292.4 |

*NOTE*: “n.d” means not detected.

**Supporting information 8: The metabolic pathways of the histidine, arginine, phenylalanine and tyrosine as well as the proteins involved in these pathways**

**Figure. S9 The metabolic pathway of histidine.** Blue part stands for proteins involved in this pathway

**Table. S11 The proteins involved in histidine metabolic pathway**

| **Name** | **Definition** | **Protein ID** | **Corresponding mRNA ID** |
| --- | --- | --- | --- |
| hisG | ATP phosphoribosyltransferase | Hirsutella_09943 | Hirsutella_09943 |
| HIS4 | phosphoribosyl-ATP pyrophosphohydrolase | Hirsutella_05555 Hirsutella_07249 | Hirsutella_05555 Hirsutella_07249 |
| hisA | phosphoribosylformimino-5-aminoimidazole carboxamide ribotide isomerase | Hirsutella_01574 | Hirsutella_01574 |
| hisF | cyclase | Hirsutella_05993 | Hirsutella_05993 |
| hisB | imidazoleglycerol-phosphate dehydratase | Hirsutella_03317 | Hirsutella_03317 |
| hisC | histidinol-phosphate aminotransferase | Hirsutella_02214 | Hirsutella_02214 |
| E3.1.3.15B | histidinol-phosphatase | Hirsutella_04399  Hirsutella_07495 | Hirsutella_04399 Hirsutella_07495 |

**Figure. S10 The metabolic pathway of arginine.** Blue part stands for proteins involved in this pathway

**Table. S12 The proteins involved in arginine metabolic pathway**

| **Name** | **Definition** | **Protein ID** | **Corresponding mRNA ID** |
| --- | --- | --- | --- |
| GOT1 | aspartate aminotransferase | Hirsutella_01419 Hirsutella_04750 | Hirsutella_07331 Hirsutella_04750 Hirsutella_01419 |
| GOT2 | aspartate aminotransferase | Hirsutella_09958 | Hirsutella_09958 |
| GPT | alanine transaminase | Hirsutella_00698 | Hirsutella_00698 |
| argJ | glutamate N-acetyltransferase | Hirsutella_03316 | Hirsutella_03316 |
| ARG56 | N-acetyl-gamma-glutamyl-phosphate reductase | Hirsutella_00053 | Hirsutella_00053 |
| E2.6.1.11 | acetylornithine aminotransferase | Hirsutella_01773 | Hirsutella_01773 |
| argE | acetylornithine deacetylase | Hirsutella_02800 | Hirsutella_08220 Hirsutella_02799 Hirsutella_02800 |
| argJ | glutamate N-acetyltransferase | Hirsutella_03316 | Hirsutella_03316 |
| OTC | ornithine carbamoyltransferase | Hirsutella_08439 | Hirsutella_08439 Hirsutella_05183 |
| argG | argininosuccinate synthase | Hirsutella_04175 | Hirsutella_04175 |
| argH | argininosuccinate lyase | Hirsutella_06202 | Hirsutella_06202 |
| E3.5.3.1 | arginase | Hirsutella_04321 | Hirsutella_04322  Hirsutella_05042 Hirsutella_04321 Hirsutella_05041 |

**Figure. S11 The metabolic pathway of phenylalanine and tyrosine.** Blue part stands for the proteins involved in this pathway

**Table. S13 The proteins involved in phenylalanine and tyrosine metabolic pathway**

| **Name** | **Definition** | **Protein ID** | **Corresponding mRNA ID** |
| --- | --- | --- | --- |
| E2.5.1.54 | 3-deoxy-7-phosphoheptulonate synthase | Hirsutella_00821 Hirsutella_08700 | Hirsutella_00821  Hirsutella_08700 |
| ARO1 | pentafunctional AROM polypeptide | Hirsutella_08845 | Hirsutella_08605  Hirsutella_08604  Hirsutella_08845 |
| aroD | 3-dehydroquinate dehydratase I | Hirsutella_03998 | Hirsutella_03998  Hirsutella_00417 |
| aroC | chorismate synthase | Hirsutella_01851 | Hirsutella_01851 |
| E5.4.99.5 | chorismate mutase | Hirsutella_02273 | Hirsutella_02273 |
| pheA2 | prephenate dehydratase | Hirsutella_00445 | Hirsutella_00445 |
| TYR1 | prephenate dehydrogenase | Hirsutella_05079 | Hirsutella_05079 |
| GOT1 | aspartate aminotransferase | Hirsutella_01419 Hirsutella_04750 | Hirsutella_07331 Hirsutella_04750 Hirsutella_01419 |
| GOT2 | aspartate aminotransferase | Hirsutella_09958 | Hirsutella_09958 |
| ARO8 | aromatic amino acid aminotransferase I | Hirsutella_05434 Hirsutella_10038 | Hirsutella_10038  Hirsutella_05434  Hirsutella_08219 |
| hisC | histidinol-phosphate aminotransferase | Hirsutella_02214 | Hirsutella_02214 |

**Supporting information 9: The proteins involved in the biosynthetic pathway of fatty acids.**

**Table. S14 The proteins involved in the biosynthesis pathway of fatty acids**

| **Name** | **Definition** | **Protein ID** | **Corresponding mRNA ID** |
| --- | --- | --- | --- |
| FAS1 | fatty acid synthase subunit beta | Hirsutella_09888  Hirsutella_06115  Hirsutella_09348  Hirsutella_09347 | Hirsutella_09348  Hirsutella_09888  Hirsutella_09347  Hirsutella_06115  Hirsutella_03120 |
| 6.4.1.2 | acetyl-CoA carboxylase | Hirsutella_08913 | Hirsutella_04700  Hirsutella_08913 |
| fabD | S-malonyltransferase | Hirsutella_01354  Hirsutella_01351 | Hirsutella_01354  Hirsutella_01351 |
| fabF | 3-oxoacyl-[acyl-carrier-protein] synthase II | Hirsutella_01470  Hirsutella_08305 | Hirsutella_01470  Hirsutella_08305 |
| FAS2 | fatty acid synthase subunit alpha | Hirsutella_08976 | Hirsutella_08976 |
| fabG | 3-oxoacyl-[acyl-carrier protein] reductase | Hirsutella_08664  Hirsutella_04368  Hirsutella_08644  Hirsutella_03259  Hirsutella_05707  Hirsutella_00915  Hirsutella_05788 | Hirsutella_08644 Hirsutella_05534  Hirsutella_05809 Hirsutella_05707  Hirsutella_09712 Hirsutella_08665  Hirsutella_04368 Hirsutella_02815  Hirsutella_08120 Hirsutella_01480  Hirsutella_06945 Hirsutella_00915  Hirsutella_08102 Hirsutella_06848  Hirsutella_07858 Hirsutella_10039  Hirsutella_04525 Hirsutella_03259  Hirsutella_08664 |
| ACSL | long-chain acyl-CoA synthetase | Hirsutella_07616  Hirsutella_07613  Hirsutella_04833 | Hirsutella_06530 Hirsutella_03367  Hirsutella_01959 Hirsutella_01551  Hirsutella_06578 Hirsutella_07613  Hirsutella_10036 Hirsutella_04833  Hirsutella_01076 Hirsutella_00828  Hirsutella_07616 Hirsutella_07830  Hirsutella_07072 Hirsutella_08765  Hirsutella_04237 |

**Supporting information 10: The metabolic pathways of citrate cycle and glycolysis/gluconeogenesis, and the proteins involved in these pathways.**

**Figure. S12 The pathway of TCA cycle.** Blue part stands for proteins involved in this pathway

**Table. S15 The proteins involved in the TCA cycle**

| **Name** | **Definition** | **Protein ID** | **Corresponding mRNA ID** |
| --- | --- | --- | --- |
| PDHA | pyruvate dehydrogenase E1 component alpha subunit | Hirsutella_05701 | Hirsutella_05701 |
| PDHB | pyruvate dehydrogenase E1 component beta subunit | Hirsutella_01008 | Hirsutella_01008 |
| DLAT | pyruvate dehydrogenase E2 component | Hirsutella_05327  Hirsutella_01468 | Hirsutella_05327 Hirsutella_01468 |
| DLD | dihydrolipoamide dehydrogenase | Hirsutella_08993 | Hirsutella_06694  Hirsutella_01318 Hirsutella_08993  Hirsutella_05961 |
| PC | pyruvate carboxylase | Hirsutella_04963 | Hirsutella_04963 |
| E4.1.1.49 | phosphoenolpyruvate carboxykinase (ATP) | Hirsutella_00749 | Hirsutella_00749 |
| MDH2 | malate dehydrogenase | Hirsutella_04041  Hirsutella_01202 | Hirsutella_04041 Hirsutella_01202 |
| CS | citrate synthase | Hirsutella_07610 Hirsutella_05796 | Hirsutella_06463  Hirsutella_05796 Hirsutella_04760  Hirsutella_01180 Hirsutella_07610 |
| ACLY | ATP citrate (pro-S)-lyase | Hirsutella_04379 Hirsutella_04378 Hirsutella_04380 | Hirsutella_04379 Hirsutella_04378 Hirsutella_04380 |
| ACO | aconitate hydratase | Hirsutella_06747 | Hirsutella_06747 |
| IDH1 | isocitrate dehydrogenase | Hirsutella_05599 | Hirsutella_05599 |
| IDH3 | isocitrate dehydrogenase (NAD+) | Hirsutella_02833 | Hirsutella_02833  Hirsutella_07914 |
| OGDH | 2-oxoglutarate dehydrogenase E1 component | Hirsutella_10169 Hirsutella_04311 | Hirsutella_10169  Hirsutella_04311 |
| DLST | 2-oxoglutarate dehydrogenase E2 component | Hirsutella_00302 | Hirsutella_00302 |
| DLD | dihydrolipoamide dehydrogenase | Hirsutella_08993 | Hirsutella_06694  Hirsutella_01318 Hirsutella_08993  Hirsutella_05961 |
| LSC2 | succinyl-CoA synthetase beta subunit | Hirsutella_07411 Hirsutella_06891 | Hirsutella_07411 Hirsutella_06891 |
| LSC1 | succinyl-CoA synthetase alpha subunit | Hirsutella_00853 | Hirsutella_00853 |
| SDHA | succinate dehydrogenase (ubiquinone) flavoprotein subunit | Hirsutella_06270 Hirsutella_07211 | Hirsutella_07211  Hirsutella_06270 Hirsutella_08018 |
| SDHB | succinate dehydrogenase (ubiquinone) iron-sulfur subunit | Hirsutella_00427 | Hirsutella_00427 |
| E4.2.1.2B | fumarate hydratase | Hirsutella_00240 | Hirsutella_04041  Hirsutella_01202 |

**Figure. S13 The pathway of Glycolysis/Gluconeogenesis.** Blue part stands for proteins involved in this pathway

**Table. S16 The proteins involved in the Glycolysis/Gluconeogenesis pathway**

| **Name** | **Definition** | **Protein ID** | **Corresponding mRNA ID** |
| --- | --- | --- | --- |
| pgm | phosphoglucomutase | Hirsutella_05031 | Hirsutella_05031 |
| GPI | glucose-6-phosphate isomerase | Hirsutella_05745 Hirsutella_08675 | Hirsutella_05745 Hirsutella_08675 |
| galM | aldose 1-epimerase | Hirsutella_03840 | Hirsutella_03840  Hirsutella_06104 |
| HK | hexokinase | Hirsutella_02360 Hirsutella_02164  Hirsutella_01203  Hirsutella_00142 | Hirsutella_08364 Hirsutella_00142 Hirsutella_02360 Hirsutella_00666 Hirsutella_02164 Hirsutella_01203 |
| FBP | fructose-1,6-bisphosphatase | Hirsutella_04940 | Hirsutella_04940 |
| E5.1.3.15 | glucose-6-phosphate 1-epimerase | Hirsutella_08699 | Hirsutella_08308 Hirsutella_07356 Hirsutella_08699 |
| pfkA | 6-phosphofructokinase | Hirsutella_00059 | Hirsutella_00059 |
| 4.1.2.13 | fructose-bisphosphate aldolase | Hirsutella_07507 | Hirsutella_07507 Hirsutella_01064 |
| TPI | triosephosphate isomerase | Hirsutella_06234 | Hirsutella_06234 Hirsutella_01221 |
| GAPDH | glyceraldehyde 3-phosphate dehydrogenase | Hirsutella_06835 | Hirsutella_06835 Hirsutella_09222 |
| PGK | phosphoglycerate kinase | Hirsutella_01695 | Hirsutella_01695 |
| gpmB | probable phosphoglycerate mutase | Hirsutella_07364 | Hirsutella_08587 Hirsutella_07364 Hirsutella_00928 |
| gpmI | 2,3-bisphosphoglycerate-independent phosphoglycerate mutase | Hirsutella_06109 | Hirsutella_06109 |
| ENO | enolase | Hirsutella_07427 | Hirsutella_07427 |
| PK | pyruvate kinase | Hirsutella_08630 | Hirsutella_08630 |
| E4.1.1.19 | arginine decarboxylase | Hirsutella_00749 | Hirsutella_00749 |
| PDHA | pyruvate dehydrogenase E1 component alpha subunit | Hirsutella_05701 | Hirsutella_05701 |
| PDHB | pyruvate dehydrogenase E1 component beta subunit | Hirsutella_01008 | Hirsutella_01008 |
| PDC | pyruvate decarboxylase | Hirsutella_10054 | Hirsutella_10054 |
| DLAT | pyruvate dehydrogenase E2 component | Hirsutella_05327  Hirsutella_01468 | Hirsutella_05327 Hirsutella_01468 |
| DLD | dihydrolipoamide dehydrogenase | Hirsutella_08993 | Hirsutella_06694 Hirsutella_01318 Hirsutella_08993 Hirsutella_05961 |
| ACSS | acetyl-CoA synthetase | Hirsutella_03927 | Hirsutella_01551 Hirsutella_03927 Hirsutella_07830 Hirsutella_01076 |
| ALDH | aldehyde dehydrogenase (NAD+) | Hirsutella_04754  Hirsutella_05765  Hirsutella_08466 | Hirsutella_04754 Hirsutella_09496 Hirsutella_00231 Hirsutella_09223 Hirsutella_05765 Hirsutella_08466 Hirsutella_08261 |
| E1.2.15 | aldehyde dehydrogenase (NAD(P)+) | Hirsutella_02909  Hirsutella_05390 | Hirsutella_09496 Hirsutella_05390 Hirsutella_02909 |
| adhP | alcohol dehydrogenase | Hirsutella_04441Hirsutella_04617 | Hirsutella_04441  Hirsutella_06588 Hirsutella_01298 |
| frmA | S-(hydroxymethyl)glutathione dehydrogenase / alcohol dehydrogenase | Hirsutella_01399 | Hirsutella_08198 Hirsutella_01399 |
| AKR1A1 | alcohol dehydrogenase (NADP+) | Hirsutella_01794 | Hirsutella_01794 Hirsutella_08349 Hirsutella_06993 Hirsutella_01298 |

**Supporting information 11: The proteins with anti-oxidant activity in *O. sinensis* zjut*.***

***Table. S17 The proteins with anti-oxidant activity in O. sinensis zjut***

| **Protein_ID** | **Description** |
| --- | --- |
| Hirsutella_05062 | manganese superoxide dismutase |
| Hirsutella_00109 | Superoxide dismutase [Cu-Zn] |
| Hirsutella_02364 | Catalase |
| Hirsutella_01603 | Catalase-3 |
| Hirsutella_03716 | Catalase |
| Hirsutella_05551 | thioredoxin reductase |
| Hirsutella_05386 | mitochondrial peroxiredoxin PRX1 |
| Hirsutella_03969 | peroxiredoxin 1 variant 2 |
| Hirsutella_08003 | peroxiredoxin 5 |
